# Simultaneous Complement Response via Lectin Pathway in Retina and Optic Nerve in an Experimental Autoimmune Glaucoma Model

**DOI:** 10.3389/fncel.2016.00140

**Published:** 2016-06-01

**Authors:** Sabrina Reinehr, Jacqueline Reinhard, Marcel Gandej, Sandra Kuehn, Rozina Noristani, Andreas Faissner, H. Burkhard Dick, Stephanie C. Joachim

**Affiliations:** ^1^Experimental Eye Research Institute, University Eye Hospital, Ruhr-University BochumBochum, Germany; ^2^Department of Cell Morphology and Molecular Neurobiology, Faculty of Biology and Biotechnology, Ruhr-University BochumBochum, Germany

**Keywords:** glaucoma, C3, complement system, lectin pathway, MAC, MASP2, retinal ganglion cells, optic nerve

## Abstract

Glaucoma is a multifactorial disease and especially mechanisms occurring independently from an elevated intraocular pressure (IOP) are still unknown. Likely, the immune system contributes to the glaucoma pathogenesis. Previously, IgG antibody depositions and retinal ganglion cell (RGC) loss were found in an IOP-independent autoimmune glaucoma model. Therefore, we investigated the possible participation of the complement system in this model. Here, rats were immunized with bovine optic nerve homogenate antigen (ONA), while controls (Co) received sodium chloride (*n* = 5–6/group). After 14 days, RGC density was quantified on flatmounts. No changes in the number of RGCs could be observed at this point in time. Longitudinal optic nerve sections were stained against the myelin basic protein (MBP). We could note few signs of degeneration processes. In order to detect distinct complement components, retinas and optic nerves were labeled with complement markers at 3, 7, 14, and 28 days and analyzed. Significantly more C3 and MAC depositions were found in retinas and optic nerves of the ONA group. These were already present at day 7, before RGC loss and demyelination occurred. Additionally, an upregulation of C3 protein was noted via Western Blot at this time. After 14 days, quantitative real-time PCR revealed significantly more *C3* mRNA in the ONA retinas. An upregulation of the lectin pathway-associated mannose-serine-protease-2 (MASP2) was observed in the retinas as well as in the optic nerves of the ONA group after 7 days. Significantly more MASP2 in retinas could also be observed via Western Blot analyses at this point in time. No effect was noted in regard to C1q. Therefore, we assume that the immunization led to an activation of the complement system via the lectin pathway in retinas and optic nerves at an early stage in this glaucoma model. This activation seems to be an early response, which then triggers degeneration. These findings can help to develop novel therapy strategies for glaucoma patients.

## Introduction

Glaucoma is one of the most common causes of blindness worldwide and in 2020 about 79.6 million people will be affected (Quigley and Broman, 2006; EGS, 2014). This neurodegenerative disease leads to a loss of retinal ganglion cells (RGCs) and their axons, which induces characteristic clinical symptoms, such as gradual visual field loss. Until now, the mechanisms behind this disease have not been fully understood.

Although an elevated intraocular pressure (IOP) remains the main risk factor for glaucoma, other mechanisms are also involved. In the last few years, abnormal antibody patterns were found in sera of patients with glaucoma (Wax et al., 1994, 2001; Grus et al., 2004). These were characterized as antibodies against ocular tissue (Joachim et al., 2008) and were detected in patients with elevated IOP as well as in patients with normal tension glaucoma. Moreover, IgG autoantibody depositions were observed in the human glaucomatous retina (Gramlich et al., 2013a). All these findings lead to the question to which extent the immune system is involved in this disease.

To investigate the pathomechanisms more specifically, an IOP-independent autoimmune glaucoma model was developed (Wax et al., 2008). In this model, immunization with ocular antigens, such as heat shock protein 27 or S100B protein lead to a loss of RGCs without IOP-elevation (Joachim et al., 2009; Casola et al., 2015). Recent studies from our group revealed antibody alterations in the sera and the occurrence of IgG deposits in the retinas of immunized animals (Joachim et al., 2012). This raises the question, how these antibodies are involved in the development of glaucoma. Possibly, antibodies activate specific pathways, which lead to apoptosis, like the complement pathway, since it is known that IgGs are able to activate the complement system (Sontheimer et al., 2005; Ehrnthaller et al., 2011). This activation takes place in several diseases, including neuromyelitis optica. Here, IgG antibodies selectively bind to aquaporin-4, which subsequently triggers complement (Hinson et al., 2007; Bradl et al., 2009).

The complement system is part of the innate immune defense. It is activated via three distinct pathways. Besides the classical pathway, the lectin and the alternative pathway are able to initiate this cascade. The membrane attack complex (MAC) is formed at the end of all pathways. It generates a pore in the target cell, which leads to osmotic imbalance, resulting in cell lysis. Inappropriate complement activation plays a crucial role in many neuropathological diseases, such as multiple sclerosis (Lucchinetti et al., 2000) or Alzheimer’s disease (Rogers et al., 1992b; Fonseca et al., 2011). Complement depositions were observed in patients with glaucoma (Tezel et al., 2010) as well as in ocular hypertension (OHT) animal models (Kuehn et al., 2006, 2008; Becker et al., 2015). But, the precise role of the complement system in glaucoma remains still unclear.

The aim of this study was to determine, if complement components are altered in the retinas and optic nerves after immunization with the bovine optic nerve homogenate antigen (ONA). We investigated via which pathway the activation occurred and at which point in time it was initiated. Alterations of the pathways and their common final path were examined over time, particularly 3–28 days after immunization. Interestingly, the first significant complement activation in the retinas and the optic nerves was already noted at day 7. This indicates that the complement system represents an early and very sensitive system of neurodegeneration before neuronal cell loss.

## Materials and Methods

### Animals

All procedures concerning animals adhered to the ARVO statement for the use of animals in ophthalmic and vision research. All experiments involving animals were approved by the animal care committee of North Rhine–Westphalia, Germany.

Male Lewis rats (Charles River, Sulzfeld, Germany), 6 weeks of age, were used for the experiments and kept under environmentally controlled conditions with free access to chow and water. Detailed observations and health checks, including eye exams, were performed regularly.

### Immunizations

The preparation and immunization of ONA was carried out as previously described (Laspas et al., 2011; Joachim et al., 2013). Rats received an intraperitoneal injection with 8 mg/ml ONA. The antigen was mixed with incomplete Freund’s adjuvant (500 μl) plus 3 μg pertussis toxin (both Sigma Aldrich, St. Louis, MO, USA). The animals of the control group (Co) were injected with NaCl in Freund’s adjuvant and pertussis toxin.

Animals were sacrificed at 3, 7, 14, and 28 days after immunization.

### Retinal Ganglion Cell Counts via Flatmounts

14 days after immunization, eyes were fixed in 4% paraformaldehyde (PFA) for 1 h and then prepared as flatmounts (*n* = 6/group). The following steps were performed at 20°C on a thermo shaker (70 rpm). First, the flatmounts were blocked with 10% donkey serum and 0.5% Triton-X in PBS for 90 min. Then, they were incubated with the RGC marker Brn-3a (Nadal-Nicolas et al., 2009) (1:300; Santa Cruz, CA, USA) overnight, followed by a 2 h incubation of donkey anti-goat Alexa Fluor 488 (1:1000; Dianova, Hamburg, Germany). From each of the four flatmount arms, three photos were captured (central, middle, and peripheral) with an Axiocam HRc CCD camera on an Axio Imager M1 fluorescence microscope (Zeiss, Jena, Germany). Cells were counted using ImageJ software (NIH, USA). Group comparison was performed after transferring the data to Statistica software (V10.0; Statsoft, Tulsa, OK, USA).

### Histology of the Optic Nerve

To evaluate the myelin status of the optic nerves 3, 7, and 14 days after immunization, longitudinal sections of the optic nerves were stained against the myelin basic protein (MBP). Briefly, the sections were blocked with 10% goat serum and 0.1% Triton-X in PBS for 60 min. The primary antibody MBP (1:100; Millipore, Darmstadt, Germany) was incubated overnight. The next day, the secondary antibody goat anti-mouse Alexa Fluor 488 (1:500; Invitrogen, Darmstadt, Germany) was added for 60 min. Nuclear staining with 4′,6 diamidino-2-phenylindole (DAPI; Serva Electrophoresis, Heidelberg, Germany) was included. Negative controls were performed by using only the secondary antibody.

### Histology of Complement Components in Retinas and Optic Nerves

In order to identify the different complement components in the retina (*n* = 5–6/group) and the optic nerve (*n* = 6–8/group), specific antibodies were used for immunofluorescence staining (**Table 1**). Briefly, sections of the retina or the optic nerve were blocked with a solution containing donkey and/or goat serum and 0.1% Triton-X in PBS. The primary antibodies were incubated at room temperature overnight. Incubation with corresponding secondary antibodies was performed for 60 min. Nuclear staining with DAPI was included to facilitate the orientation on the slides. Negative controls were performed by using secondary antibodies only.

**Table 1:** Primary and secondary antibodies applied for immunohistochemistry of retinal and optic nerve tissue.

Primary antibodies				Secondary antibodies			
Antibody	Company	Tissue	Dilution	Antibody	Company	Tissue	Dilution
Brn-3a	Santa Cruz	Retina	1:100	Donkey anti-goat Alexa Fluor 488	Dianova	Retina	1:1000
C1q	Quidel	Retina	1:2500	Donkey anti-goat IgG Cy3	Abcam	Retina	1:750
C3	Cedarlane	Retina	1:500	Goat anti-rabbit IgG Cy 3	Linaris	Retina	1:500
		Optic nerve	1:500			Optic nerve	1:500
C5b-9 (MAC)	Biozol	Retina	1:100	Donkey anti-mouse Dy Light 488	Dianova	Retina	1:250
		Optic nerve	1:100	Goat anti-mouse Alexa Flour 488	Invitrogen	Optic nerve	1:500
MASP2	Biozol	Retina	1:400	Donkey anti-rabbit Alexa Fluor 555	Invitrogen	Retina	1:400
		Optic nerve	1:100			Optic nerve	1:700
MBP	Millipore	Optic nerve	1:100	Goat anti-mouse Alexa Flour 488	Invitrogen	Optic nerve	1:500

### Histological Examination of Retinas and Optic Nerves

The photographs were taken using a fluorescence microscope (Axio Imager M1). In the retina, two photos of the peripheral and two of the central part of each section were captured for each point in time. In the optic nerve, three photos were captured (proximal, middle, and distal). The images were transferred to Corel Paint Shop Pro (V13; Corel Corporation, CA, USA) and excerpts were cut out. Complement positive cells (C1q, C3, and MAC) were counted using ImageJ software.

For MBP and the mannose-associated-serine-protease 2 (MASP2) analyses we used an ImageJ macro (Joachim et al., 2014). Briefly, we first transformed the images into greyscale. After background subtraction (MBP: 50 pixel; MASP2 retina: 100 pixel; MASP2 optic nerve: 7.2 pixel) the lower and upper thresholds were set (MBP: lower: 13.86, upper: 113.18; MASP2 retina: lower: 10.29, upper: 70; MASP2 optic nerve: lower: 12.8; upper: 86). The percentage of the labeled areas was measured for each picture using the macro, exported to Excel and transferred to Statistica.

### RNA Preparation and cDNA Synthesis

For RNA preparation, retinas (*n* = 3–6/group) from every point in time were isolated, transferred into lysis buffer containing 2-mercaptoethanol (Sigma–Aldrich) and snap frozen in liquid nitrogen. Total RNA was extracted with the Gene Elute Mammalian Total RNA Miniprep Kit according to the manufacturer’s instructions and digested with RNase-free DNase I (Sigma–Aldrich). The quality and quantity of RNA were assessed by measurement of the ratio of absorbance values at 260 and 280 nm (BioSpectrometer^®^, Eppendorf, Hamburg, Germany). Total RNA (1 μg) was used for reverse transcription using a cDNA synthesis kit (Thermo Fisher Scientific, Waltham, MA, USA).

### Quantitative Real-time PCR

The designed oligonucleotides are shown in **Table 2**. Quantitative real-time-PCR (qRT-PCR; Roche Applied Science, Mannheim, Germany) technology was performed using SYBR Green I on the Light Cycler^®^ 96 (Roche Applied Science). Primer concentration was optimized to a final concentration of 200 nM and combined with 200 ng of retinal RNAs per well. We set up two reactions per RNA sample (duplicates) with a final volume of 20 μl per single reaction (Ray et al., 2005; Horvat-Brocker et al., 2008; Luft et al., 2014). Each qRT-PCR was performed in triplicate from each retina and for each point in time and repeated twice. The average threshold cycle (Ct) values of the two independent experiments were used to calculate the ratios for the primers (Pfaﬄ et al., 2002). In order to obtain amplification efficiencies of different primer sets, we generated standard curves by a twofold dilution series with template amounts ranging from 5 to 125 ng cDNA per well. The Ct values of the reference genes (β*-actin and cyclophilin*) were taken into account. For statistical evaluation of Ct variations and calculated relative expression variations, data were analyzed for significant differences by a pairwise fixed reallocation and randomization test using the REST^©^ software (Qiagen, Hilden, Germany) (Pfaﬄ et al., 2002).

**Table 2:** Sequences of oligonucleotide pairs.

Gene	Forward (F) and reverse (R) oligonucleotides	GenBank accession number	Amplicon size
β*-actin-*F β*-actin-*R	cccgcgagtacaaccttct tcaagcggtacctactgc	NM_031144.3	72 bp
*C1qa-*F *C1qa-*R	cgggtctcaaaggagagagag ctctgtacccccttagacc	NM_001008515.1	88 bp
*C1qb-*F *C1qb-*R	gcactccagggataaaagga accactcaatcctctctttccc	NM_019262.1	75 bp
*C3-*F *C3-*R	tcgaaatccctcccaagtc gtaacaggggaacttctagc	NM_016994.2	60 bp
*C5-*F *C5-*R	tctcaggccaaagagagacc ttcgacgatttatgtttgtggca	XM001079130.4	73 bp
*Cyclophilin-*F *Cyclophilin-*R	tgctggaccaaacacaaatg tcgtacaccagaaacccttc	M19553.1	88 bp
*MASP2-*F *MASP2-*R	gctggaagatacactacacaagca gtggattaccagtgtaaagtgg	NM_172043.1	76 bp

*The listed primer pairs were used in quantitative real-time PCR experiments, while β*-actin* and *cyclophilin* served as housekeeping genes. The predicted amplicon sizes are given. Abbreviations: F, forward; R, reverse; bp, base pair.*

### Quantitative Western Blot Analysis

Seven days after immunization, retinas (*n* = 4–6/group) were used for Western Blot analyses. The proteins were isolated by mechanical and chemical methods. First, the frozen retinas were homogenized with a metal homogenizer (Neolab, Heidelberg, Germany). Then, 150 μl of a lysis buffer (RIPA buffer; Cell signaling technology, Cambridge, UK) combined with protease inhibitory solution (Sigma–Aldrich) was added. The retina solution was treated with ultrasound. Thereafter, the RIPA buffer was allowed to react on ice for 50 min. The last existing cell components were separated by centrifugation for 30 min (13200 rpm, 4°C). The protein concentration was determined by a commercial bicinchoninic acid assay (BCA; Thermo Fisher Scientific). 20 μg per sample was loaded per lane of a 4–12% Bis-Tris gel (NuPAGE, Invitrogen). After the blotting step using the NuPAGE Transfer buffer (60 min, 200 V), the nitrocellulose membranes were blocked with a mixture of 5% milk powder in a PBS/0.05% Tween-20 solution. The primary antibodies C3 (1:500; Cedarlane, Burlington, ON, Canada), MASP2 (1:1000; Biozol, Eching, Germany), and α-tubulin (1:20000; Sigma–Aldrich) were used for the protein detection. The secondary antibodies were labeled with fluorochromes like Alexa Fluor 680 (donkey anti-rabbit, 1:5000; Invitrogen) and DyLight 800 (donkey anti-mouse, 1:2000; Thermo Scientific). The protein bands were recorded and analyzed with the Odyssey infrared imager system 2.1 (LI-COR Bioscience, Lincoln, NE, USA). The cleavage products of C3, namely iC3bα and iC3bβ, were recorded together (63 and 75 kDa), while the uncleaved C3α chains were recorded separately (110 kDa). MASP2 was recorded at 72 kDa. The protein signal intensity was normalized to the reference protein α-tubulin, which was recorded at 50 kDa.

### Statistics

Regarding immunohistology and Western Blot, data are presented as mean ± standard error (SEM), unless otherwise noted. The ONA group was compared to the Co group via two-tailed Student’s *t*-test using Statistica Software. Regarding qRT-PCR, data are presented as median ± quartile + minimum + maximum and were assessed using REST software. *P*-values below 0.05 were considered statistically significant.

## Results

### No Early Effects on Retinal Ganglion Cells

**FIGURE 1:**
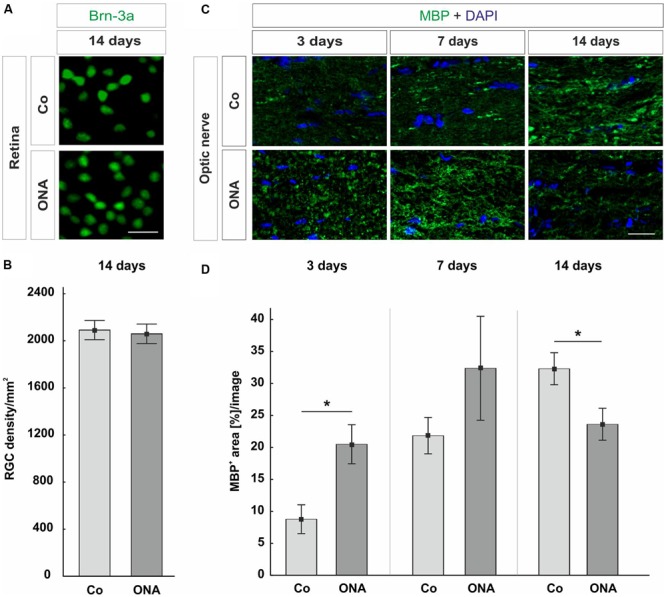
**(A)** Representative photos of Brn-3a stained flatmounts of the ONA and control group (Co). Both groups showed a similar number of Brn-3a^+^ cells. **(B)** The RGC counts revealed no difference between the two groups (*p* = 0.9). **(C)** Optic nerve sections labeled with MBP (green) at 3, 7, and 14 days. Cell nuclei were visualized with DAPI (blue). **(D)** At 3 days, a significant larger MBP^+^ area was measured in the ONA group compared to Co (*p* = 0.02). After 7 days, the expression of MBP went back to control level (*p* > 0.05). A significant decrease in the MBP^+^ area was observed in the ONA group at 14 days (*p* = 0.03). Values are mean ± SEM. Scale bars: 20 μm.

No changes in the ganglion cell density could be observed in the ONA group compared to control group at 14 days (*p* = 0.96; **Figures 1A,B**). Later, at 28 days, a significant RGC loss has been described in the ONA animals (Laspas et al., 2011).

### Demyelination Processes in the Optic Nerves

A possible optic nerve demyelination was analyzed via immunohistological staining against MBP at 3, 7, and 14 days (**Figures 1C,D**). At 3 days, a significantly larger MBP^+^ area was measured in ONA optic nerves compared to Co (*p* = 0.02). After 7 days, the MBP area went back to the control values (*p* > 0.05). Later on, at 14 days, a decrease of MBP^+^ area could be observed (*p* = 0.03).

### Complement Activation in the Retina and Optic Nerve after Immunization

To evaluate if the complement system is activated over time, the factors C3 and MAC were immunohistochemically analyzed in retinas 3, 7, 14, and 28 days after immunization. Additionally, expression patterns of retinal C3 and C5, as part of the MAC complex, were quantified via qRT-PCR at 3, 7, and 14 days. Western Blot analyzes were performed for C3 at 7 days. Optic nerves were stained against the protein C3 and MAC 3, 7, and 14 days after immunization.

**FIGURE 2:**
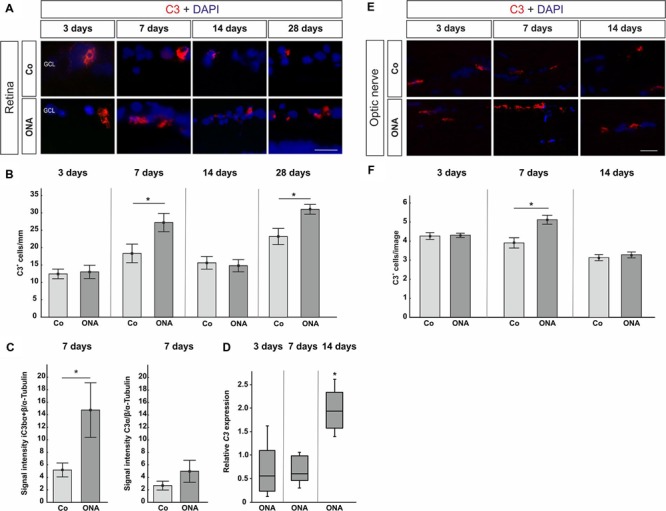
**(A)** Exemplary photos of C3 (red) and DAPI (blue) labeled retinas 3, 7, 14, and 28 days after immunization. **(B)** At 3 days, no difference in the number of C3^+^ cells could be detected (*p* > 0.05). Significantly more C3 depositions were noted in the ONA group after 7 days (*p* = 0.002). At 14 days, no difference was observed (*p* > 0.05). Significantly more C3 depositions were noted in the ONA group 28 days after immunization (*p* = 0.003). **(C)** Protein levels of iC3bα+β and C3α at 7 days analyzed via Western Blot. In the ONA group, significantly more iC3bα+β was observed (*p* = 0.049). The C3α chains were not altered (*p* > 0.05). **(D)** Expression levels of *C3* at 3, 7, and 14 days measured with qRT-PCR. After 3 and 7 days, no changes in *C3* expression could be noted (*p* > 0.05). The quantification of *C3* revealed a significant upregulation in the ONA group at 14 days (*p* = 0.011). **(E)** To evaluate C3 in the optic nerve, sections were stained with C3 (red) and DAPI (blue) 3, 7, and 14 days after immunization. **(F)** No alterations in C3^+^ cells were noted after 3 days (*p* > 0.05). At 7 days, significantly more C3^+^ cells could be observed in ONA optic nerves (*p* = 0.02). After 14 days, the number of C3^+^ cells was not altered anymore (*p* > 0.05). Abbreviations: GCL, ganglion cell layer. Values for immunostaining and Western Blot are mean ± SEM. Values for qRT-PCR are median ± quartile ± maximum/minimum. Scale bars: 20 μm.

Concerning the C3 expression in the retina (**Figures 2A–D**), at 3 days, no differences were detected in the ONA group (*p* > 0.05). Also, no changes in *C3*-mRNA expression were observed at this point in time (*p* > 0.05). Significantly more C3 depositions were found in ONA retinas at 7 days (*p* = 0.002). Additionally, Western Blot analysis revealed an upregulation of iC3bα and iC3bβ (*p* = 0.049), while no alteration was measured for the C3α chains (*p* > 0.05). The mRNA expression levels of retinal *C3* were not altered at 7 days (*p* > 0.05). 14 days after immunization, no difference in immunostaining of C3 could be observed in the ONA group (*p* > 0.05), while mRNA quantification demonstrated a significant upregulation of *C3* in ONA retinas (*p* = 0.011). 28 days after immunization the number of C3^+^ cells in the retina was increased in the ONA group (*p* = 0.03).

In the optic nerves, no changes for C3 were noted after 3 days (*p* > 0.05). At 7 days, significantly more C3 depositions were found in ONA optic nerves (*p* = 0.02). Later on, at 14 days, no more differences in regard to C3 staining could be seen (*p* > 0.05; **Figures 2E,F**).

**FIGURE 3:**
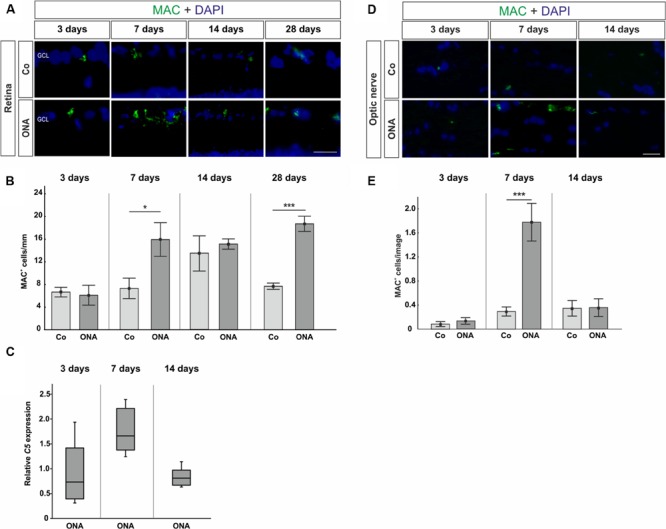
**(A)** Retinas were labeled with MAC (green) and DAPI (blue) and MAC^+^ cells were counted in the retina at all points in time. **(B)** After 3 days, no difference in the number of MAC^+^ cells could be noted (*p* > 0.05). At 7 days, the number of MAC^+^ cells in the ONA group was significantly increased (*p* = 0.03). At 14 days, no changes were noted (*p* > 0.05), while at 28 days again significantly more MAC depositions were observed in the ONA group (*p* = 0.003). **(C)** Quantitative RT-PCR analysis revealed no differences in *C5* expression, which is part of the MAC complex (*p* > 0.05). **(D)** Sections of the optic nerves were stained with MAC (green) and DAPI (blue) at all points in time. **(E)** Three days after immunization, no alterations in MAC depositions could be noted (*p* > 0.05). A significant increase of MAC^+^ cells was observed in ONA optic nerves at 7 days (*p* = 0.001). After 14 days, no changes in the number of MAC^+^ cells were noted anymore (*p* > 0.05). Values for immunostaining are mean ± SEM. Values for qRT-PCR are median ± quartile ± maximum/minimum. Abbreviations: GCL, ganglion cell layer. Scale bars: 20 μm.

Regarding MAC staining in the retina (**Figures 3A,B**), at 3 days, no alterations were detected in the ONA animals (*p* > 0.05). 7 days after immunization more MAC^+^ cells were observed in the ONA group (*p* = 0.03). At 14 days, ONA animals presented control levels (*p* > 0.05). At 28 days, again significantly more MAC^+^ cells were noted in ONA retinas (*p* = 0.003). No alterations in expression levels of *C5* mRNA could be noted in ONA retinas at any point in time (3 days: *p* = 0.3; 7 days: *p* = 0.08; 14 days: *p* = 0.18; **Figure 3C**).

The staining of MAC in the optic nerves showed no changes after 3 days (*p* > 0.05), while significantly more MAC depositions were detected in the optic nerves of the ONA animals at day 7 (*p* = 0.001). After 14 days, no alterations regarding MAC expression could be noted anymore (*p* > 0.05; **Figures 3D,E**).

In conclusion, complement activation is present in retinas and optic nerves of ONA animals at 7 days (**Tables 3–5**).

**Table 3:** (A,B) Histological detection of complement factors in the retina.

(A)
**Retina**	**3 days**	***P*-value**	**7 days**	***P*-value**	**14 days**	***P*-value**

**C3^+^ cells/mm**						
Co	12.44 ± 1.39		18.38 ± 2.67		15.64 ± 1.81	
ONA	13.02 ± 1.92	0.81	27.25 ± 2.64	**0.04**	14.80 ± 1.75	0.75
**MAC^+^ cells/mm**						
Co	6.66 ± 0.85		7.31 ± 1.81		13.49 ± 3.09	
ONA	6.11 ± 1.76	0.78	15.94 ± 2.98	**0.03**	15.15 ± 0.92	0.62
**C1q^+^ cells/mm**						
Co	0.29 ± 0.19		12.28 ± 2.65		9.20 ± 0.93	
ONA	0.80 ± 0.46	0.33	12.61 ± 3.59	0.94	7.02 ± 1.65	0.28
**MASP2^+^ area [%]/image**						
Co	1.29 ± 0.45		6.03 ± 1.75		7.91 ± 2.08	
ONA	1.49 ± 0.66	0.81	13.49 ± 2.64	**0.04**	4.63 ± 1.17	0.21

**(B)**

**Retina**	**28 days**	***P*-value**

**C3^+^ cells/mm**		
Co	23.25 ± 2.33	
ONA	31.06 ± 1.40	**0.03**
**MAC^+^ cells/mm**		
Co	7.89 ± 0.56	
ONA	18.72 ± 1.34	**0.003**

*Values are mean ± SEM. Significant values are marked in bold.*

**Table 4:** Histological detection of complement components in the optic nerve.

Optic nerve	3 days	*P*-value	7 days	*P*-value	14 days	*P*-value
**C3^+^ cells/image**						
Co	4.26 ± 0.18		4.18 ± 0.29		3.13 ± 0.16	
ONA	4.30 ± 0.11	0.98	5.49 ± 0.25	**0.02**	3.27 ± 0.15	0.79
**MAC^+^ cells/image**						
Co	0.08 ± 0.04		0.29 ± 0.08		0.35 ± 0.13	
ONA	0.13 ± 0.05	0.78	1.78 ± 0.31	**0.0004**	0.36 ± 0.15	0.99
**MASP2^+^ area [%]/image**						
Co	6.11 ± 1.56		4.32 ± 0.43		2.63 ± 0.70	
ONA	8.30 ± 1.11	0.38	7.26 ± 0.46	**0.04**	2.28 ± 0.49	0.91

*Values are mean ± SEM. Significant values are marked in bold.*

**Table 5:** Analyses of complement components via quantitative real-time PCR.

	3 days	7 days	14 days
β*-actin*	1.09	1.09	1.31
*Cyclophillin*	0.92	0.92	0.76
*C1qa*	1.19 (0.48–3.35)	0.91 (0.58–1.22)	0.71 (0.58–0.90)
*P*-value	0.61	0.73	0.07
*C1qb*	1.03 (0.33–2.71)	0.70 (0.49–1.06)	0.8 (0.50–1.51)
*P*-value	0.84	0.22	0.59
*C3*	0.55 (0.24–1.09)	0.61 (0.46–0.99)	1.94 (1.58–2.34)
*P*-value	0.16	0.25	**0.011**
*C5*	0.73 (0.39–1.42)	1.66 (1.37–2.12)	0.81 (0.67–0.98)
*P*-value	0.29	0.08	0.18
*MASP2*	1.03 (0.65–1.63)	0.57 (0.28–0.89)	1.61 (1.04–1.31)
*P*-value	0.92	0.17	0.09

*Values are median + SE range. Significant values are marked in bold.*

### Activation through the Lectin Pathway

To identify the pathway of complement activation in the retinas, analyses of the classical pathway, namely C1q, and the lectin pathway, namely MASP2, were performed at 3, 7, and 14 days. Expression patterns of retinal *C1qa, C1qb*, and *MASP2* were analyzed via qRT-PCR 3–14 days after immunization. At 7 days, MASP2 was also investigated through Western Blot. Evaluation of the lectin pathway in the optic nerves was also undertaken via immunohistology. Sections were therefore labeled with an antibody against MASP2.

**FIGURE 4:**
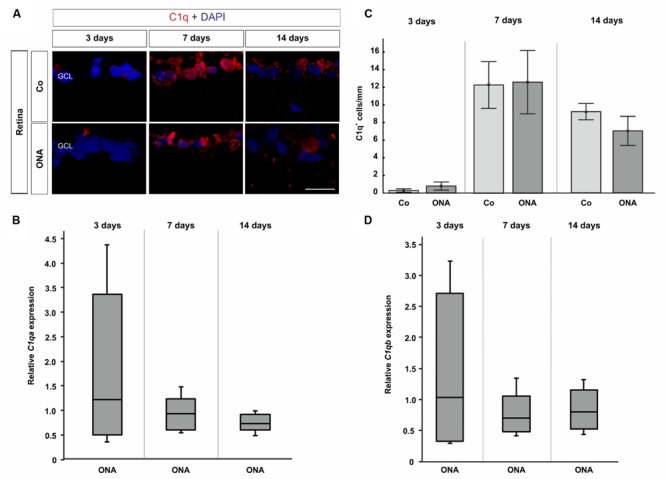
**(A)** Exemplary photos of retinas stained with C1q (red) and DAPI (blue). **(C)** 3 days after immunization no difference between the ONA group and controls was detectable (*p* > 0.05). Also, no changes could be noted at 7 and 14 days (*p* > 0.05). **(B)** The quantification via qRT-PCR revealed no expression changes after 3, 7, and 14 days in regard to *C1qa* (*p* > 0.05). **(D)** Also, comparable *C1qb* expression levels were noted in the ONA group at 3, 7, and 14 days (*p* > 0.05). Abbreviations: GCL, ganglion cell layer. Values for immunostaining are mean ± SEM. Values for qRT-PCR are median ± quartile ± maximum/minimum. Scale bar: 20 μm.

Regarding C1q in the retina, no difference could be observed via histology at all points in time (*p* > 0.05; **Figures 4A,C**). The quantification for *C1qa* revealed no changes in mRNA expression levels at 3, 7, and 14 days (*p* > 0.05; **Figure 4B**). Also, no alterations in the expression of *C1qb* could be observed via qRT-PCR (*p* > 0.05; **Figure 4D**).

**FIGURE 5:**
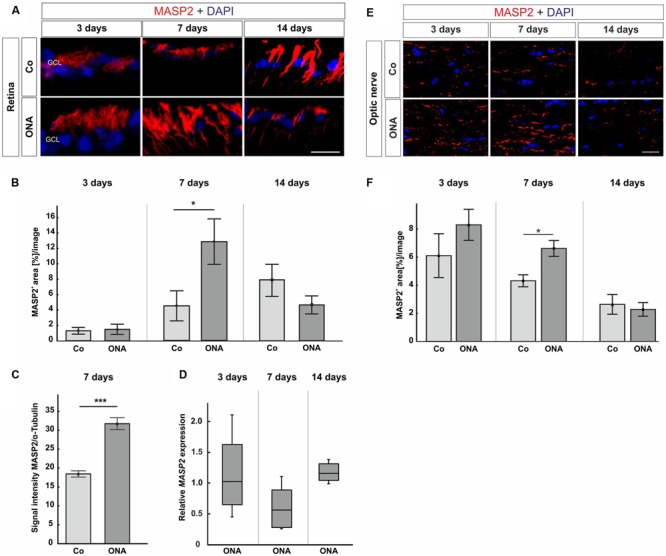
**(A)** Photos from retinas stained with MASP2 (red) and DAPI (blue) at 3, 7, and 14 days followed by area analysis. **(B)** Three days after immunization, no alterations in MASP2^+^ area could be noted in the ONA group (*p* > 0.05). An increased MASP^+^ area was noted in ONA retinas at 7 days (*p* = 0.04). At 14 days, the MASP2^+^ area went back to control levels in the ONA group (*p* > 0.05). **(C)** At 7 days, protein analyses of MASP2 through Western Blot revealed an upregulation in the ONA group (*p* < 0.001). **(D)** No altered *MASP2* expression could be noted at all points in time via qRT-PCR (*p* > 0.05). **(E)** Optic nerve sections were labeled with MASP2 (red) and cell nuclei with DAPI (blue) 3, 7, and 14 days after immunization. **(F)** The MASP2^+^ area analysis revealed no changes in the ONA group 3 days after immunization. After 7 days, a significantly larger MASP2^+^ area was noted in ONA optic nerves (*p* = 0.009). At 14 days, the expression went back to control values (*p* > 0.05). Abbreviations: GCL, ganglion cell layer. Values for immunostaining and Western Blot are mean ± SEM. Values for qRT-PCR are median ± quartile ± maximum/minimum. Scale bars: 20 μm.

In the retinas, no differences in MASP2 staining (**Figures 5A,B**) were observed in the ONA group at 3 days (*p* > 0.05). Then, at 7 days, a larger MASP2^+^ area was seen in the ONA group (*p* = 0.04). Protein analyses via Western Blot also confirmed a significant increase of MASP2 in ONA retinas at 7 days (*p* < 0.001; **Figure 5C**). After 14 days, no alterations in the MASP2^+^ area could be observed (*p* > 0.05). No changes of the *MASP2* mRNA level could be measured at 3, 7, and 14 days (*p* > 0.05; **Figure 5D**).

Concerning MASP2 expression in the optic nerves, at 3 days, the MASP2 area analysis revealed no changes in the ONA animals (*p* > 0.05; **Figures 5E,F**). Significantly more MASP2 was noted in the ONA group after 7 days (*p* = 0.0009). At 14 days, the expression of MASP2 went back to control levels (*p* > 0.05).

These data suggest that the complement system is simultaneously activated in the retinas and the optic nerves via the lectin pathway in this autoimmune glaucoma model (**Tables 3–5**).

## Discussion

It is known that antibodies against RGCs can lead to a reduction of cells, when injected intravitreally, and that these antibodies interact directly with the RGCs (Kornguth et al., 1982; McCall et al., 1987).

Based on the findings of autoantibodies in glaucoma, a contribution of the complement system seems to be likely. Therefore, we performed immunohistochemistry, qRT-PCR, and Western Blot analyses of several components of the complement pathway. Our results demonstrate, for the first time, a complement activation in this autoimmune glaucoma model. Increased levels of complement proteins were already shown in sera of patients with primary open-angle glaucoma (POAG). Furthermore, alterations in complement proteins could be found in retinal samples of the same patients (Boehm et al., 2010). In animal models of OHT, an increase of complement components was also observed. For instance, a significant upregulation of C1q, C3, and MAC was described 14 and 28 days after OHT-induction in animals with considerably increased IOP (Kuehn et al., 2006). Our group could show that even a moderate increase of IOP leads to an activation of the complement components C3 and MAC (Becker et al., 2015). Another OHT-study could demonstrate that a depletion of the complement system reduces the loss of RGCs due to inhibition of intrinsic and extrinsic apoptotic pathways (Jha et al., 2011). Importantly, our results indicate that the complement activation occurs independently from IOP. A significant increase of C3 and MAC was observed in the retinas and the optic nerves 7 days after immunization in the autoimmune glaucoma model. This leads to the question, how the activation is actually initialized. It is possible that IgG antibodies, which were previously observed in patients with POAG, trigger the complement system (Gramlich et al., 2013a). Although the authors stated that they could not find a correlation of complement proteins and IgG accumulations, an involvement of the complement system in the pathogenesis of glaucoma cannot be excluded (Gramlich et al., 2013a,b). In a previous study, IgG autoantibodies were detected in the retinas and the optic nerves of an autoimmune glaucoma model 2 and 4 weeks after immunization (Laspas et al., 2011). Based on these findings we assumed that the complement system is activated via the classical pathway through C1q in our model. Interestingly, an increase of C1q was neither detected in histology nor in qRT-PCR analysis over time. This is contrary to results in human glaucoma eyes and models of OHT, where a C1q upregulation was noted (Kuehn et al., 2006; Stasi et al., 2006; Tezel et al., 2010). In our IOP-independent model, the activation via C1q seems to play a subordinate role. It is possible that 2 weeks after immunization only a small number of autoantibodies were present and that hence, we could not detect any classical pathway activation. We do not exclude that at subsequent points in time C1q could trigger the complement system in this model. Further studies should be performed to investigate the role of C1q in degeneration processes more precisely. However, it is also possible that other components lead to an activation of the complement system. In Alzheimer’s disease, the aggregated amyloid protein is a strong complement activator (Rogers et al., 1992a; Veerhuis et al., 2003). These β-amyloid (Aβ) plaques can also be found in glaucoma models (McKinnon et al., 2002) and drugs targeting Aβ would be promising for glaucoma therapy (Guo et al., 2007; Salt et al., 2014).

Interestingly, we detected an activation of the lectin pathway already 7 days after immunization. MASP2 forms a complex with the mannose binding lectin (MBL). MBL itself is able to bind to a wide range of microorganisms, including bacteria, viruses, parasites, and fungi (Neth et al., 2000). But not solely cells displaying carbohydrate structures on their surfaces can activate the lectin pathway. It is known that hypoxia induces alterations in the cell surface of endothelial cells which could activate the complement system via the lectin pathway (Collard et al., 1999). It is also reported that MBL is able to bind to apoptotic and necrotic cells (Ogden et al., 2001; Turner, 2003; Stuart et al., 2005). Another study showed a proinflammatory role for the lectin pathway-mediated complement activation after myocardial ischemia-reperfusion in rats (Jordan et al., 2001). This could be an evidence that this pathway plays a role in other inflammatory pathobiologies. In eye diseases, like age-related macular degeneration, the dysregulation of the complement system plays a crucial role in the pathogenesis. Here, the activation is antigen-independent and triggered via the lectin and the alternative pathway (Gehrs et al., 2006; Frederick and Kleinman, 2014). Further evidence that complement activation in glaucoma could be initiated via the lectin pathway is provided by a study by Tezel et al. (2010). Here, proteomic analyses of human donor glaucoma eyes revealed an upregulation of proteins that are linked to the lectin pathway. All these results support our assumption that the complement system is mainly activated via the lectin pathway in glaucoma.

The activation of the complement system in the retina and optic nerve raises the question how these components could enter the eye, which is known to be immune privileged (Medawar, 1948; Taylor, 2009). Here, it is reasonable to assume that invading microglia cells could be a producer of complement proteins. They represent the macrophage population of the central nervous system (Kettenmann et al., 2011). In the retina, microglia are mainly located in the ganglion cell layer or in the inner plexiform layer. In the optic nerve activated microglia are first localized in the optic nerve head (Bosco et al., 2011). Microglia are known to be involved in many neuroinflammatory processes, for example in multiple sclerosis (Jiang et al., 2014), where the microglia activation is also associated with RGC death in an experimental autoimmune encephalomyelitis model (Horstmann et al., 2013). They were also observed in human glaucomatous retina (Gramlich et al., 2013a) as well as in the retina and optic nerve head of glaucoma models (Bosco et al., 2008; Laspas et al., 2011; Joachim et al., 2012, 2014). Recent studies reported that microglia are a source of retinal complement. After light-induced damage, Rutar et al. identified C3 expression by microglia, which lead to the suggestion that these cells were responsible for the local spreading of complement in the retina (Rutar et al., 2011). *In vitro* experiments indicated that microglia cells synthesized complement components, like C1q, C3, C5, and MASP1 (Luo et al., 2011).

In summary, we demonstrated a contribution of the complement system in an IOP-independent autoimmune glaucoma model. This activation occurred prior to RGC death and optic nerve degeneration and is mainly triggered through the lectin pathway. Most interestingly, the complement cascade is simultaneously activated in the retina and in the optic nerve. These results lead to the assumption that complement activation triggers cell death in glaucoma and could therefore help to develop new therapies to delay glaucoma progression as well as to recognize glaucoma disease at early points in time, before neurodegeneration is evident.

## Author Contributions

SR performed experiments, analyzed data, and wrote the manuscript; JR analyzed data and revised the manuscript; MG performed experiments and analyzed data; SK and RN performed experiments; AF and HD revised the manuscript; SJ designed the study and revised the manuscript. All authors have read and approved the final manuscript.

## Conflict of Interest Statement

The authors declare that the research was conducted in the absence of any commercial or financial relationships that could be construed as a potential conflict of interest.
